# Completion of autobuilt protein models using a database of protein fragments

**DOI:** 10.1107/S0907444911039655

**Published:** 2012-03-16

**Authors:** Kevin Cowtan

**Affiliations:** a Department of Chemistry, University of York, Heslington, York YO10 5DD, England

**Keywords:** model building, databases, *Buccaneer*

## Abstract

Two developments in the process of automated protein model building in the *Buccaneer* software are presented. A general-purpose library for protein fragments of arbitrary size is described, with a highly optimized search method allowing the use of a larger database than in previous work. The problem of assembling an autobuilt model into complete chains is discussed. This involves the assembly of disconnected chain fragments into complete molecules and the use of the database of protein fragments in improving the model completeness. Assembly of fragments into molecules is a standard step in existing model-building software, but the methods have not received detailed discussion in the literature.

## Abstract 2

Two developments in the process of automated protein model building in the *Buccaneer* software are described: the use of a database of protein fragments in improving the model completeness and the assembly of disconnected chain fragments into complete molecules.

## 1. Background

This paper outlines two developments of relevance to the problem of automated protein model building. The initial application of these techniques in the *Buccaneer* model-building software is presented. The developments are the following.

(i) An efficiently searchable database of protein fragments which may be used for diverse purposes including the con­version of a C^α^ trace to main-chain models, the building of missing loops and termini, and the correction of residue insertions and deletions. This library has been implemented for loop building in the *Coot* software (Emsley *et al.*, 2010 ▶), as well as for applications in automated model building described here.
(ii) The automated ‘tidying’ of a fragmentary autobuilt protein model, with the aim of reducing the manual effort required to complete the model. Automated model building sometimes leads to models which may consist of multiple disconnected fragments, especially at low resolution or when disordered loop regions are not visible in the electron density. These fragments must be assembled into one or more molecules, which may involve the application of symmetry operators and cell translations to some of the fragments. In the case of noncrystallographic symmetry (NCS) it is also necessary to assign the fragments to different copies of the molecule.

### 1.1. Use of databases of protein fragments

The use of databases of protein fragments in the determination and validation of atomic models is well established in both manual and automated model building.

Kleywegt & Jones (1996 ▶) described the use of pentapeptide fragments in the program *OOPS* for the validation of the protein backbone trace.

Jones & Thirup (1986 ▶) used a database of pentapeptides in the reconstruction of a main-chain trace from C^α^ positions alone, although Payne (1993 ▶) claimed better results using force fields. Esnouf (1997 ▶) used a library of 16 533 hexapeptide fragments in the same way to obtain main-chain coordinates which matched the refined X-ray structure to a high precision.

Terwilliger (2003 ▶) employed a library of tripeptide fragments to extend existing fragments of protein chain by adding additional residues at the N- or C-terminus and Sheldrick (2010 ▶) used tripeptides to find initial protein fragments. Joosten *et al.* (2008 ▶) used a library of pentapeptide fragments in a similar way to build missing loops in protein structures.

The development described here recognizes the success of these methods and describes an efficient method for building and searching a library of protein fragments of arbitrary length (bounded by some chosen value). The database is optimized for very fast homology searches, allowing the use of a much larger database than in previous work. The use of a much larger database also provides the potential to perform searches restricted by residue-type filters without compromising coverage beyond usefulness.

### 1.2. Tidying and completion of protein models

Automated model building typically produces as an intermediate result a set of protein-chain fragments, some of which may have been docked into the protein sequence. Ultimately, these will need to be assembled into molecules. A problem arises in determining how the fragments are connected to one another. When protein molecules are tightly packed together the molecule boundaries may not be obvious, and as a result it is possible to link fragments which belong not to the same chain but rather to symmetry-related chains. If the density for the link is obvious, this step may be performed by automatic or manual model completion; however, this is often not the case.

The problem becomes more complex in the case of non­crystallographic symmetry (NCS). In this case, the fragments must also be assigned to the correct NCS copy of the molecule, as well as to the correct asymmetric unit. The problem may be further complicated in the case of hetero-oligomers (protein complexes consisting of heterogeneous sequences), although this is mainly a bookkeeping problem.

Various approaches to model tidying are implemented in the main automated model-building packages [for example, *ARP*/*wARP* (Cohen *et al.*, 2004 ▶) and *RESOLVE* (Terwilliger, 2003 ▶)], with the details varying according to the model-building algorithm and the information available; however, the details have not been widely discussed in the literature. This paper presents the model-tidying steps implemented in the *Buccaneer* software from v.1.5.

### 1.3. The *Buccaneer* software for automated model building

The *Buccaneer* software is used for automatic interpretation of protein structures on the basis of the electron-density map (Cowtan, 2006 ▶, 2008 ▶). The calculation is iterative, with multiple cycles of model building interspersed with occasional refinement steps using *REFMAC* (Murshudov *et al.*, 2011 ▶) to improve the current model and electron density. The steps involved in a single cycle of model building are as follows.

(i) Finding C^α^ atoms: candidate C^α^ positions are located by searching the electron density for likely features.
(ii) Growing fragments: the candidate C^α^ atoms (or input chains) are grown by adding residues at either end, guided by the electron density and constrained by the allowed region of the Ramachandran plot.
(iii) Joining fragments: overlapping fragments are joined to make longer chains.
(iv) Linking fragments: nearby N- and C-termini are examined to see if they can be linked by inserting one or two additional residues.
(v) Assigning sequence: likelihood comparison between the density of each residue in the work structure and the density from residues of a reference structure is used to identify the likelihood of each residue being of a particular type. Comparison with the known sequence allows longer fragments to be matched to the sequence.
(vi) Correcting sequence: insertions and deletions in the model as identified in the sequence-assignment step are corrected by rebuilding to add or delete a residue where possible.
(vii) Filtering fragments in poor density: residues which have not been docked into the sequence and are in poor density are removed.
(viii) Building NCS: any NCS relationships found in the model are used to extend existing chains by combining all of the NCS-related chains.
(ix) Pruning fragments: fragments which provide inconsistent interpretations of the same electron density are examined. The poorer fragment is removed.
(x) Rebuilding: side-chain atoms and carbonyl O atoms are added to the model.

This process is repeated over several cycles. In subsequent cycles, the finding step is modified to preferentially find C^α^ positions which are in regions where no model is present.

## 2. A library of protein fragments

A library of real protein fragments of arbitrary length is employed to interpret electron density and correct existing models. In order to support both interactive graphical model building (where users demand immediate feedback) and automated model building (where many possible model fragments may need to be tested to match a particular feature), it must be possible to perform a very rapid search for fragments containing some atoms matching a desired conformation.

For example, to fit the main-chain atoms to a C^α^ trace the database will be searched for all six-peptide fragments matching the C^α^ atoms surrounding a particular peptide bond and the peptide atoms from the middle peptide of the best-fitting fragment will be used to provide the main-chain atoms for that peptide group. Similarly, to build a missing loop in a protein structure a search will be performed for all fragments for which the initial and final pairs of C^α^ atoms in the fragment may be superimposed on the last two C^α^ atoms before the break and the first two C^α^ atoms after the break.

A library has therefore been constructed using the 500 well refined protein structures of the Richardsons’ ‘Top 50’ database (Lovell *et al.*, 2003 ▶), excluding residues for which the temperature factors of the C^α^ atoms exceed 40 Å^2^. This provides a database of 106 295 amino acids in 1327 continuous fragments. For each amino acid, the residue type and the coordinates of the N, C^α^ and C atoms are stored (in turn providing sufficient information to locate the C^β^ and O atoms). The entire database is stored as a single list of amino-acid records.

The most frequent type of search which will be performed on the database is to find all fragments for which some (possibly discontinuous) set of C^α^ atoms superpose well on the C^α^ atoms of some search fragment. The search fragment is in turn provided as a list of amino-acid records, with null records inserted as placeholders to represent residues for which the location is unknown. Thus, to search for a missing loop of four residues, an eight-residue search fragment is constructed from the two residues before the missing loop, four null residues and the two residues after the missing loop.

Performing a least-squares superposition for every fragment in the database would be computationally demanding, so an initial pre-selection phase is performed to produce a subset of fragments which may be good matches to the search fragment. This pre-selection involves a computationally cheaper distance-matrix score.

**Figure 1:**
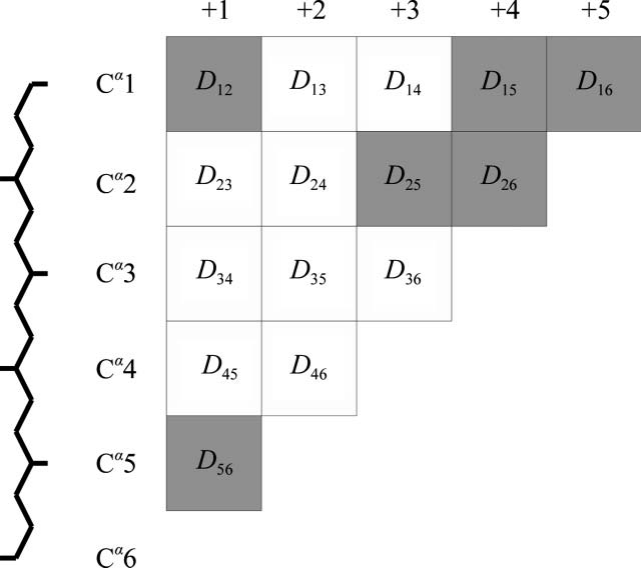
Running distance-matrix representation of a single fragment, where *D*_*ij*_ is the distance between the *i*th and *j*th C^α^ atoms. The shaded cells are those available for loop fitting using only two C^α^ atoms at each end of the fragment.

In order to minimize the computational overhead, distance matrices for the search fragment and for the database are precalculated. For the search fragment, a triangular matrix is calculated with the first row giving the distances from the first C^α^ to the remaining *n* − 1, the second row the distances from the second C^α^ to the remaining *n* − 2 and so on. The columns of this matrix correspond to the diagonals of the upper triangle of a conventional distance matrix (illustrated in Fig. 1 ▶). If an atom is missing, the distance is set to a negative flag value.

**Figure 2:**
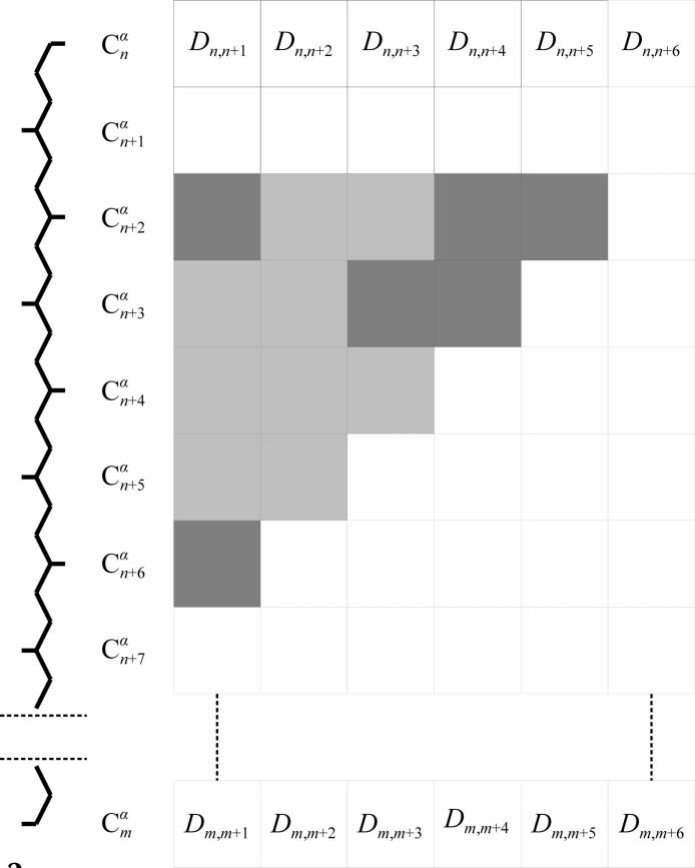
Running distance-matrix representation of the protein-chain database, where *D*_*i*,*j*_ is the distance between the *i*th and *j*th C^α^ atoms. The shaded cells are those which would be used to score the fit of a search fragment against a particular range in the database.

For the database of *n*_db_ residues, an *n*_db_ × 20 rectangular ‘running distance matrix’ is pre-calculated, with each row giving distances from the first C^α^ to the following 20, thus representing fragments of up to 21 residues. This is illustrated in Fig. 2 ▶ for a reduced width of six residues. Any distances which span chain boundaries are set to the flag value.

In order to identify a set of possibly matching fragments, all that needs to be done is to compare the non-missing values in the fragment distance matrix to the corresponding values obtained by starting from each row of the database distance matrix in turn. A sum of squared differences is used to identify likely matches.

To further optimize the calculation, the sum-of-squares calculation may be terminated early as soon as the sum exceeds a threshold value. The threshold value is controlled by a parameter which determines how many matches will be returned and is updated regularly by sorting the current list of matches, truncating to the desired number and setting the threshold to the value of the worst remaining match.

The limitation of the distance-matrix score is that the distance matrix of a set of coordinates is invariant under inversion of these coordinates through a centre of symmetry, and so the initial search also returns fragments which are the inverse of the search fragment. The resulting list of candidate fragments must therefore be re-scored using a full least-squares superposition and r.m.s. difference calculation. The resulting list is resorted according to the r.m.s. difference.

For some purposes it may be desirable to restrict the search to fragments for which the sequence obeys some criterion, for example to take into account the different main-chain conformations which can occur around Gly or Pro. This is achieved by allowing a mask of 20 binary digits to be set for each position in the search fragment, indicating which of the 20 amino-acid types are allowed to appear at that position in the fragment. This provides an additional restriction on the search results which may be evaluated by simple logical operations.

## 3. Automated model tidying

The steps employed in the completion of the atomic model in the current version of *Buccaneer* are as follows.

(i) The various fragments built by the chain-tracing and sequence-docking algorithms are grouped into discontinuous chains using a scoring function that rewards compactness and penalizes sequence duplication. This removes a tedious manual step of assigning chain IDs and renaming the resulting chain fragments by hand.
(ii) Where there are discontinuities (or breaks) in the resulting chains, an attempt is made to fix these discontinuities by pruning any overlap and placing a fragment from a stored database of protein fragments across the gap.

The steps involved in the grouping of fragments into chains are described in detail in §§[Sec sec3.1]3.1, 3.2 and 3.3. The correction of breaks is discussed in §[Sec sec3.4]3.4. These steps are inserted between steps (ix) and (x) of the workflow described in §[Sec sec1.3]1.3.

### 3.1. Grouping fragments into chains

The process of grouping fragments into chains involves assigning a chain identifier to each fragment such that the fragments which make up a single chain all have the same chain identifier. Furthermore, the resulting fragments may need to be transformed by the application of crystallographic symmetry elements to form a compact molecule.

In the simplest case of a single sequence with no noncrystallographic symmetry (NCS), the process of allocating chain identifiers is simply a matter of separating a set of fragments which comprise a single complete chain from those which are incorrectly built or sequenced (however, the remaining fragments are retained with dummy chain identifiers in case they contain correctly located but wrongly sequenced residues).

The general case involves two additional layers of complexity. Firstly, there may be multiple copies of the molecule in the asymmetric unit. In this case, multiple chains with different chain identifiers must be built and each fragment must be allocated to one of the chains in such a way as to build several compact molecules. Secondly, in the case of a hetero-complex there may be multiple distinct sequences involved.

The basic steps of the calculation are as follows.

(i) In the case where multiple sequences are present, those fragments which have been docked to one of the sequences are sorted according to which sequence was used. Each sequence is then considered in turn and the following steps are applied to all the fragments belonging to that sequence.
(ii) A set of ‘seed’ fragments are identified by the method described in §[Sec sec3.2]3.2, including one fragment from each NCS copy of the molecule. The fragments are chosen such that they all incorporate some common range of sequence numbers and thus must belong to distinct copies of the molecule. The selection of this range is made in such a way as to maximize the number of NCS copies identified, subject to the validation criteria described below.
(iii) The seed fragments are then grown by successively adding an additional fragment to a seed by the method described in §[Sec sec3.3]3.3. Each fragment is scored for its geometrical proximity to each seed (taking into account crystallographic symmetry) and penalized for any sequence overlap with that seed. The fragment which obtains the highest score to be docked to a seed is then added to that seed. The calculation repeats until all fragments have been assigned or the highest score fails to reach a threshold.

Steps (ii) and (iii) are repeated for each sequence until all sequences have been considered. The fragments are then assembled into chains by grouping all the fragments sharing a chain identifier in order of sequence number. In some cases, sequence numbers of grouped fragments may overlap; in this case, insertion codes are used to ensure that each residue is uniquely identified.

### 3.2. Identification of seed fragments

The identification of ‘seed’ fragments is performed as follows. Firstly, a matrix is constructed whose order is the number of fragments under consideration. The matrix is used to store flags identifying which fragments overlap. For each pair of sequences, the number of residues of overlap is identified. If the overlap exceeds 12 residues *and* the overlapped regions have similar conformations, the number of overlapped residues is stored in the matrix. (In this context, a similar conformation is identified by the least-squares superposition of the best-matched 50% of the overlapped C^α^ coordinates having an r.m.s. difference of less than 1 Å.)

A depth-first permutation search is then performed to identify the largest subset of fragments all of which overlap. There will usually be multiple equal solutions; in this case, the set is chosen for which the total number of residues in the overlapping fragments is the greatest.

At first glance the algorithm is computationally expensive, since potentially 2^*n*^ sets must be considered, where *n* is the number of fragments. In practice, the number of overlapped sequences does not significantly exceed the number of NCS copies and depth searches may be terminated early if they cannot match the current best solution; thus, in practice the computational cost of this step is negligible.

The fragments thus selected contain the same sequence of residues in a similar conformation and thus can be assumed to be different NCS copies of one part of the structure. Each of the selected seed fragments is therefore allocated a different chain identifier and becomes the core of that chain.

### 3.3. Allocation of additional fragments to the chains

This step is performed iteratively. Every unallocated fragment is considered and the score is calculated for adding that fragment to each chain. The highest scoring chain/fragment combination is selected and the fragment is added to that chain. This will affect all subsequent scores for that chain and therefore the calculation is then repeated from the start.

The scoring function rewards geometrical compactness and penalizes sequence inconsistencies as follows. Each C^α^ atom within 5 Å of a C^α^ atom which has already been allocated to a given chain provides a score of +1 for adding the fragment to that chain. Each residue which has been docked into sequence with a sequence number clashing with a residue already allocated to a given chain provides a score of −2 for adding the fragment to that chain.

In this way, fragments which are intimate to an existing chain but which do not contain the same set of sequence are added to that chain. The process continues until no positive scores remain.

### 3.4. Correction of chain breaks

Often it will occur that there are gaps in the trace of the protein chain. These most commonly occur for one of two reasons.

(i) Flexible surface loops for which the electron density is poor.
(ii) Mistracings where the chain trace has left the chain (often following a side chain or disulfide bridge) and the chain trace is then continued in a subsequent fragment.

For the gap to be corrected, any wrongly traced residues (*e.g.* following a side chain or disulfide bridge) must first be removed by pruning back at least enough residues to remove any duplicated sequence numbers from the ends of the two fragments (multiple choices about how many residues to prune from each end are possible and additional pruning may be required to eliminate all mistraced residues, so multiple prunings are tested) and then selecting a fragment from a database of protein-chain fragments to bridge the gap.

Note that caution is required in this step. Earlier in the *Buccaneer* calculation an attempt is made to link spatially proximal N- and C-termini without regard to sequence. Sometimes these linkages are made incorrectly. However, this mistake is not serious, because when docking the resulting chain to the sequence the two parts of the joined chain will usually dock to different places in the sequence, at which point the error can be corrected by breaking the chain again. When linking chains on the basis of previously assigned sequences, the use of the sequence to validate the link is no longer available, so mistakes introduced at this stage will never be corrected. As a result, it was found to be necessary to limit the maximum length of the bridging fragment to six amino acids (*i.e.* two amino acids overlap with each chain and a maximum of two amino acids of gap). Longer missing loops must still be built manually. Since the errors arise from the presence of wrongly sequenced fragments which occur early in the model-building process when the fragments are short, this constraint should probably be relaxed to allow longer loops to be built once the model is approaching completion, at which point errors become less likely.

### 3.5. Additional applications of the fragment database

Two existing steps in the *Buccaneer* calculation were also rewritten to make use of the fragment database. The ‘linking’ step (joining nearby N- and C-termini irrespective of sequence) and ‘correction’ step (correcting insertions and deletions by rebuilding one or three residues with two residues) both made use of a routine for building a loop of two residues by searching over allowed Ramachandran angles. Both of these steps have been replaced by an equivalent implementation using the fragment database.

## 4. Results

Some preliminary results are presented here on the applicability of the fragment database and on the automated model-tidying features in the *Buccaneer* software.

### 4.1. Coverage as a function of fragment size in the fragment database

To investigate the usefulness of the fragment database, an exhaustive search was performed to test for a given fragment length how well each fragment in the database can be represented by some other fragment from the database.

Each possible fragment of the chosen length was extracted from the database in turn and used as a search model to find other similar fragments. In every case the best-fitting fragment will be the original fragment, so the best fit is discarded and the second-best match is used. Two statistics are calculated for the matching fragment: the r.m.s. deviation between the C^α^-­atom positions and those of the search fragment, and the distance between the worst-matching C^α^ atom and the corresponding atom in the search fragment. This calculation was performed for fragments of six, nine and 12 residues (as would be used in fitting missing loops of two, five and eight residues, respectively).

**Figure 3:**
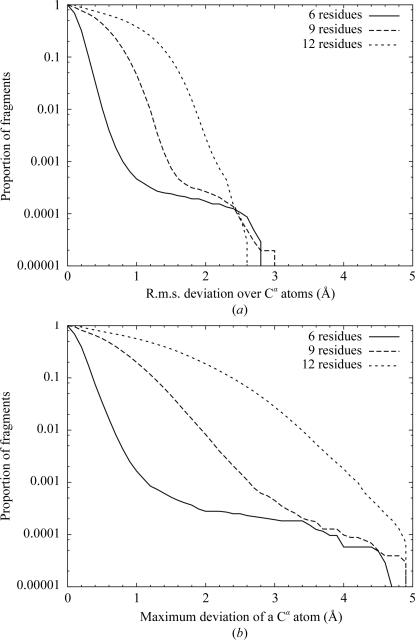
Tail plot of the proportion of search fragments for which the fit of the best-matching fragment is worse than a given criterion for different fragment lengths. (*a*) R.m.s. deviation of C^α^ positions between the best database fragment and the search fragment; (*b*) maximum deviation of any C^α^ positions between the best database fragment and the search fragment.

The results are shown in Fig. 3 ▶ as tail plots showing the proportion of the search fragments for which the difference from the database fragment is no worse than a given value.

The r.m.s. deviations are worse than 1.0 Å for 0.04% of six-residue fragments, 5% of nine-residue fragments and 38% of 12-residue fragments. Given that a significant proportion of the fragments in the database will be in very similar helical or strand conformations, this suggests that the library will be of limited use for 12-residue fragments except for common motifs.

Similarly, the worst deviating atom has a displacement of worse than 1.5 Å for 0.05% of six-residue fragments, 5% of nine-residue fragments and 36% of 12-residue fragments. (Note the change in distance criterion compared with the previous data.) This again suggests that 12-residue fragments will be of less use, since automated refinement is likely to struggle to correct errors of this magnitude.

As a result, the database provides effectively complete coverage for fragments of up to six residues or for loop fitting over only two missing residues. (This case was previously handled by a simple Ramachandran search; however, the database approach has the advantage of providing a computationally cheaper sampling of conformation space which increases in density as the frequency of that conformation increases.)

For missing loops of intermediate length (3–6 residues), the database will provide good loop conformations in a subset of cases where the loop happens to match one in the database and so will catch common turn motifs, for example. For longer loops, the database is likely to be useful only in less frequent cases. However, this approach has been shown to have good success by Choi & Deane (2010 ▶) for loops of up to 20 residues with a larger database of structures.

### 4.2. Automated model tidying in the *Buccaneer* software

The model-tidying procedure was applied to the same 55 test structures used in Cowtan (2008 ▶) and is detailed in the supplementary material of that paper; the data were obtained from the JCSG (Joint Center for Structural Genomics, 2006 ▶). Of the resulting models, 29 contained fragments which were grouped into chains by the tidying algorithm. Some of these structures included multiple NCS copies of the structure and therefore the total number of chains assembled was 50.

Each of the 50 tidied chains was examined to determine the proportion of the chain corresponding to a single molecule in the final structure. As the model becomes more complete, the assignment becomes easier, so these proportions are tabulated along with the completeness of the chain in Table 1 ▶.

In every case where the chain is at least 60% complete, at least 80% is correctly assigned to a single molecule and in 44 of 48 such cases the assignment is entirely correct or correct apart from a few trailing residues. For the two cases where the completeness is less than 50%, the grouping of fragments into chains is rather less accurate.

**Figure 4:**
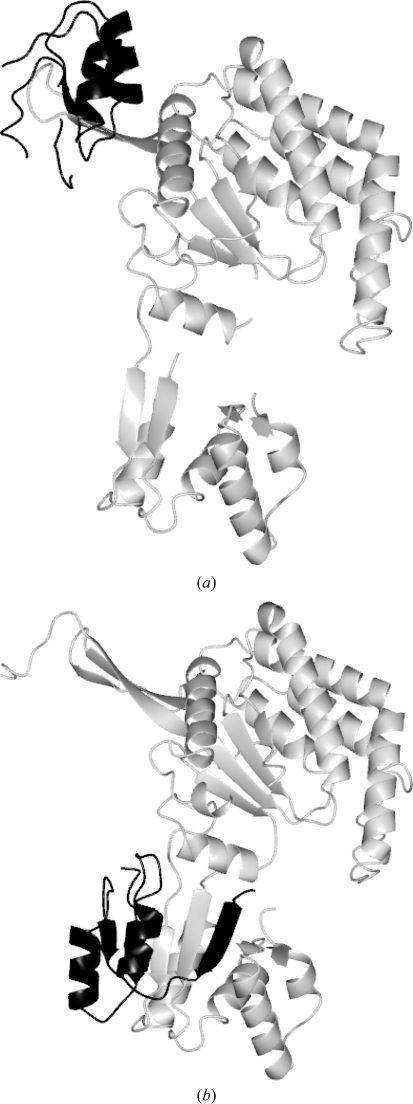
Partially incorrect assembly of the model for 1vlu from multiple fragments. The wrongly positioned region is shown in black (*a*) in the *Buccaneer* model and (*b*) in the deposited structure.

The case of the 1vlu *A* chain (as labelled by *Buccaneer*; this is actually the *B* chain in the deposited structure) is shown in Fig. 4 ▶, in which 91% of the chain has been built but only 83% of the residues built correspond to a single molecule. In this case the deposited model contains chain breaks and the *Buccaneer* model shows chain breaks in similar positions. The disconnected range of residues 331–391 has been placed at the wrong end of the molecule. It is probable that the error could have been corrected in this specific case by adding a term rewarding proximity of sequence number to the scoring function; however, this was not tested because in the experience of the author the incorrect linking of chains across protein contacts is a significant problem in the early stages of building and this problem is likely to be exacerbated by such a change.

### 4.3. Application of the fragment database in the *Buccaneer* software

The usefulness of the fragment database in automated building was tested by rewriting two existing steps of the *Buccaneer* calculation to make use of the database and by adding a new loop-building step using the database, as described in §[Sec sec4.2]4.2. The results of these changes were tested individually and in combination.

The results of the model-building calculation are rather sensitive to changes in the algorithm or input data, so to determine whether each change made an improvement multiple model-building runs were used. For each of the 55 test structures used in Cowtan (2008 ▶) ten model-building runs were performed using ten different sets of free reflections for both model building and refinement. The change in the set of reflections used to calculate the initial map is sufficient to significantly alter the results of the first model-building step and the differences propagate to subsequent cycles.

The percentage of the model built and correctly sequenced (measured by the percentage of residues built with the correct residue type and with the C^α^ within 1.9 Å of the correct position) was averaged over the 550 runs to obtain a score for this method.

Furthermore, the entire set of calculations was then repeated using lower resolution data. For these calculations, the data resolution was truncated by 0.4 Å, the *B* factor was increased by 20 Å^2^ and the density-modification step (using the *Parrot* software; Cowtan, 2010 ▶) was rerun on the truncated data. The resolutions of the original data sets vary over the range 1.4–3.2 Å and the truncated data over the range 1.8–3.6 Å.

The results of these calculations are shown in Table 2 ▶. The first step modified (‘link’) is the linking of chain fragments irrespective of sequence [step (iii) in the *Buccaneer* calculation], the next (‘correct’) is the correction of insertions and deletions during sequencing [step (v) in the *Buccaneer* calculation]. These steps were previously performed using an exhaustive search over allowed Ramachandran angles, in the first case to build a link of up to two residues and in the second to rebuild a stretch of either one or three residues with two residues. Finally, a new loop-building step was added, similar to the ‘link’ step but performed after the sequence has been assigned to the chains. Unlike the ‘link’ step, the loop-building step may prune an arbitrary number of residues from either chain to bring similarly numbered residues into proximity.

The updated link step makes minimal difference to the amount of model built, but does provide a speed benefit over the previous (Ramachandran search) implementation. The updated correct step gives a small improvement in the amount of model built, although the difference is comparable to the noise among different runs. The loop-building step shows no significant improvement in the proportion built. It is a recurring problem in the development of the model-building algorithm that the improvements are marginal and hard to distinguish from noise, even with the large number of test runs. However, in each of four cases where only the correct step is changed the results always improve, suggesting that this result is significant.

However, the benefit of the loop-building step can be seen in the connectivity of the model, which is a benefit when it comes to finishing the model by hand. The number of fragments in the output model gives an indication of what is happening. For the original version, the average number of fragments over the 550 autobuilt models is 8.7; when the loop-building step is added, this reduces to 7.4 (similar changes are seen when combining the loop-building step with the other new steps and when the resolution is truncated). A reduction in the number of fragments without a reduction in the proportion built implies an improvement in connectivity. The implication is that the loop-building step is most commonly dealing with cases where chains are coming into close proximity but failing to meet (and possibly branching down side chains) rather than true loop-building problems when there are missing residues.

To summarize, using the fragment database for the link step reduces the computational overhead, using the fragment database for the correct step provides a small improvement in completeness and using the fragment database for loop building provides a significant improvement in connectivity.

### 4.4. Other applications of the fragment library

The fragment library has also been used in the implementation of a loop-building tool, *Sloop*, which is capable of building short missing loops in incomplete protein models. As noted above, the usefulness of this tool varies according to whether the loop concerned happens to conform to an existing motif.

A tool for converting a C^α^ trace into a main-chain (polyalanine) trace has also been implemented. The results show similar high levels of accuracy to those of Esnouf (1997 ▶). The program has not been released owing to the availability of many other tools for this task; however, the source code is available from the author on request.

The use of the library for the building and validation of motifs in the *Coot* graphical model-building and validation software (Emsley *et al.*, 2010 ▶) is under development.

### 4.5. Discussion

The tidying of fragments into chains is an important element of an automated model-building calculation, principally because it reduces the manual intervention required later in the structure-solution process. The technique described here is reliable when the completeness of the model is good and is completely general with respect to NCS and hetero-complexes, without requiring knowledge of the number of copies of a given sequence present in the asymmetric unit.

The protein-fragment database is capable of reproducing the various functionalities implemented by previous authors, with the efficient search algorithm allowing the use of a larger database than in previous implementations. Some preliminary applications have been explored and a range of future applications are planned, including the following.

(i) Use of the loop-building code to build longer loops when the model is nearly complete. This may be in a single step, or possibly using the stepwise approach of Joosten *et al.* (2008 ▶) where a suitable large fragment is not found in the library.
(ii) Use of the fragment library to rebuild regions of the chain where residue type influences geometry, in particular in the vicinity of Gly and Pro residues.
(iii) Testing the use of a subset of the fragment library to replace the current Ramachandran search in the chain-growing step in *Buccaneer*, in a manner similar to that of Terwilliger (2003 ▶).
(iv) Use of the fragment library to provide validation scores in the manner of Jones & Thirup (1986 ▶) in the *Coot* software.
(v) Extension of the fragment-database concept to handle nucleotides.

## Figures and Tables

**Table 1:** Reliability of the model-tidying algorithm as measured by the proportion of each autobuilt chain corresponding to a single chain in the deposited structure

Structure (chain)	Proportion belonging to a single chain (%)	Chain completeness (%)
1vjn (*A*)	72	49
1zej (*B*)	75	46
1z85 (*A*)	81	90
1vlu (*B*)	81	73
1vlu (*A*)	83	91
1zej (*A*)	92	69
1vr8 (*A*)	95	99
1vp7 (*C*)	97	100
1vk3 (*A*)	98	90
41 cases	100	62–100

**Table 2:** Proportion of models built and correctly sequenced with different building strategies; results are averaged over 550 runs on 55 structures Values in parentheses are standard deviations across the ten runs of 55 structures.

	Full resolution		Truncated resolution	
Method	Percentage built	No. of chains	Percentage built	No. of chains
Original version	86.2 (0.6)	8.7 (0.4)	75.1 (1.0)	13.0 (0.4)
DB for link	86.2 (0.5)	8.6 (0.6)	75.5 (0.9)	12.8 (0.5)
DB for correct	86.5 (0.6)	8.7 (0.3)	76.2 (1.3)	12.9 (0.7)
DB for loop build	86.1 (0.4)	7.4 (0.4)	75.3 (0.9)	11.4 (0.4)
DB for link, correct	86.6 (0.7)	8.6 (0.3)	76.4 (0.6)	12.6 (0.6)
DB for link, correct, loop build	86.6 (0.7)	7.3 (0.3)	76.5 (0.9)	11.1 (0.7)

## References

[bb1] Choi, Y. & Deane, C. M. (2010). *Proteins*, **78**, 1431–1440. 10.1002/prot.22658 20034110

[bb2] Cohen, S. X., Morris, R. J., Fernandez, F. J., Ben Jelloul, M., Kakaris, M., Parthasarathy, V., Lamzin, V. S., Kleywegt, G. J. & Perrakis, A. (2004). *Acta Cryst.* D**60**, 2222–2229. 10.1107/S0907444904027556 15572775

[bb3] Cowtan, K. (2006). *Acta Cryst.* D**62**, 1002–1011. 10.1107/S0907444906022116 16929101

[bb4] Cowtan, K. (2008). *Acta Cryst.* D**64**, 83–89. 10.1107/S0907444907033938 PMC2394793 18094471

[bb5] Cowtan, K. (2010). *Acta Cryst.* D**66**, 470–478. 10.1107/S090744490903947X PMC2852311 20383000

[bb6] Emsley, P., Lohkamp, B., Scott, W. G. & Cowtan, K. (2010). *Acta Cryst.* D**66**, 486–501. 10.1107/S0907444910007493 PMC2852313 20383002

[bb7] Esnouf, R. M. (1997). *Acta Cryst.* D**53**, 665–672. 10.1107/S0907444997005829 15299854

[bb8] Joint Center for Structural Genomics (2006). *JCSG Data Archive.* http://www.jcsg.org/datasets-info.shtml.

[bb9] Jones, T. A. & Thirup, S. (1986). *EMBO J.* **5**, 819–822. 10.1002/j.1460-2075.1986.tb04287.x PMC1166864 3709525

[bb10] Joosten, K., Cohen, S. X., Emsley, P., Mooij, W., Lamzin, V. S. & Perrakis, A. (2008). *Acta Cryst.* D**64**, 416–424. 10.1107/S0907444908001558 PMC2467521 18391408

[bb11] Kleywegt, G. J. & Jones, T. A. (1996). *Acta Cryst.* D**52**, 829–832. 10.1107/S0907444996001783 15299648

[bb12] Lovell, S. C., Davis, I. W., Arendall, W. B., de Bakker, P. I., Word, J. M., Prisant, M. G., Richardson, J. S. & Richardson, D. C. (2003). *Proteins*, **50**, 437–450. 10.1002/prot.10286 12557186

[bb13] Murshudov, G. N., Skubák, P., Lebedev, A. A., Pannu, N. S., Steiner, R. A., Nicholls, R. A., Winn, M. D., Long, F. & Vagin, A. A. (2011). *Acta Cryst.* D**67**, 355–367. 10.1107/S0907444911001314 PMC3069751 21460454

[bb14] Payne, P. W. (1993). *Protein Sci.* **2**, 315–324. 10.1002/pro.5560020303 PMC2142385 8453371

[bb15] Sheldrick, G. M. (2010). *Acta Cryst.* D**66**, 479–485. 10.1107/S0907444909038360 PMC2852312 20383001

[bb16] Terwilliger, T. C. (2003). *Acta Cryst.* D**59**, 38–44. 10.1107/S0907444902018036 PMC2745878 12499537

